# The development of an eye movement-based deep learning system for laparoscopic surgical skills assessment

**DOI:** 10.1038/s41598-022-15053-5

**Published:** 2022-08-15

**Authors:** R. J. Kuo, Hung-Jen Chen, Yi-Hung Kuo

**Affiliations:** 1 Department of Industrial Management, National Taiwan University of Science and Technology, Taipei, Taiwan; 2 Department of Data Science, Soochow University, No. 70, Linhsi Road, Shihlin District, Taipei City, 111 Taiwan; 3 Department of Marketing and Distribution Management, National Kaohsiung University of Science and Technology, No.1, University Road, Yanchao District, Kaohsiung City, 82445 Taiwan; 4 Department of New Product Introduction, Solid State Storage Technology Corporation, Hsinchu City, Taiwan

**Keywords:** Human behaviour, Classification and taxonomy, Data mining, Machine learning

## Abstract

The development of valid, reliable, and objective methods of skills assessment is central to modern surgical training. Numerous rating scales have been developed and validated for quantifying surgical performance. However, many of these scoring systems are potentially flawed in their design in terms of reliability. Eye-tracking techniques, which provide a more objective investigation of the visual-cognitive aspects of the decision-making process, recently have been utilized in surgery domains for skill assessment and training, and their use has been focused on investigating differences between expert and novice surgeons to understand task performance, identify experienced surgeons, and establish training approaches. Ten graduate students at the National Taiwan University of Science and Technology with no prior laparoscopic surgical skills were recruited to perform the FLS peg transfer task. Then k-means clustering algorithm was used to split 500 trials into three dissimilar clusters, grouped as novice, intermediate, and expert levels, by an objective performance assessment parameter incorporating task duration with error score. Two types of data sets, namely, time series data extracted from coordinates of eye fixation and image data from videos, were used to implement and test our proposed skill level detection system with ensemble learning and a CNN algorithm. Results indicated that ensemble learning and the CNN were able to correctly classify skill levels with accuracies of 76.0% and 81.2%, respectively. Furthermore, the incorporation of coordinates of eye fixation and image data allowed the discrimination of skill levels with a classification accuracy of 82.5%. We examined more levels of training experience and further integrated an eye tracking technique and deep learning algorithms to develop a tool for objective assessment of laparoscopic surgical skill. With a relatively unbalanced sample, our results have demonstrated that the approach combining the features of visual fixation coordinates and images achieved a very promising level of performance for classifying skill levels of trainees.

## Introduction

The development of valid, reliable, and objective methods of skills assessment is central to modern surgical training. Despite the shortened training time for trainees with the advent of working time directives^1,2^, the increased awareness of iatrogenic injuries and errors has enhanced the importance for surgeons to demonstrate proficiency and meet competency requirements^3^.

In the past, surgical assessment relied on informal skill acquisition and the advancement of the apprenticeship model; various tools are now available to surgical trainers, and numerous rating scales have been developed and validated for quantifying surgical performance^4,5^. However, many of these scoring systems are potentially flawed in their design in terms of reliability. Effective assessment depends on the availability and presence of a reviewer trained in the assessment methodology^6^. The ultimate goal of surgical assessment is to develop systems that are both objective and independent. Defining surgical skills using objective metrics, such as path length or number of movements, has been achieved to some extent in laparoscopic surgery^7^. However, this is still largely limited to training settings where computer-based metrics are recorded from virtual reality simulators^8^.

The eye-tracking technique, which provides a more objective investigation of the visual-cognitive aspects of the decision-making process^9^, has been proposed as a potential assessment tool that is not limited by some of the restrictions of laparoscopic metric measurements. For this reason, eye tracking techniques have recently been utilized in surgery domains for skill assessment and training^10^, and their use has been focused on investigating differences between expert and novice surgeons to understand task performance, identify experienced surgeons, and establish training approaches^11^.

Differences in eye movement patterns between experts and novices across a range of possible measurements have been reported in previous studies. Experts have been found to spend more time fixating on the target than on the instrument itself during instrument movements^12–14^. They spend more time fixating on the vitals monitor during a simulated operation^15–17^ and display differences in general eye movement statistics, such as lower saccadic rates and higher peak velocities^18^, independent of the targets of these eye movements. These results suggest the possibility of using eye movements as a method of assessing surgical skills. However, the above studies compared experts and novices, but they did not examine surgeons at intermediate levels, which would provide insights into how eye movement patterns develop with experience.

The studies that focused on assessing surgeons’ skills used machine learning algorithms to distinguish between experts and novices^19–22^. The basic idea is to input a set of eye-movement data into a computer program and adjust the parameters of the program to obtain an optimal classification of surgeons in terms of skill or experience, mostly a binary distinction between experts and novices. For example, Ahmidi, et al.^21^ demonstrated that the Markov Model and k-means clustering analysis could discriminate skill levels and tasks with classification accuracies of 82.5% and 77.8%, respectively, by incorporating eye and instrument movements.

Excellent classification performance was reported by Richstone, et al.^19^, who used linear discriminate analysis (LDA) and nonlinear neural network analyses (NNA) to classify surgeons into experts and non-experts based on their performances during live transperitoneal laparoscopic renal surgeries and simulator tasks. Based on the findings, LDA and NNA were able to accurately distinguish experts and non-experts with 91.9% and 92.9% accuracy, respectively, in the simulated surgical environment and 81.0% and 90.7%, respectively, in the live surgical environment.

The use of various machine learning techniques and eye-movement parameters across different studies has shown that eye-movement data can be used to distinguish between experts and novices as well as surgical stages across a range of tasks and eye-movement parameters. Since current medical educators rely on subjective measurements of surgical skills, eye metrics can be used as the basis for objective assessment in future surgical education and certification. Further development of this potential educational tool and assessment of its ability to reliably classify a larger group of surgeons and track the progress of surgical skills during postgraduate training are necessary^11,19^.

The movement of the eyes is typically recorded as a time series of gaze point coordinates from both eyes. Once these data are acquired, it must be processed to extract important information and used to draw conclusions about the proficiency of the trainees with the implementation of supervised machine learning algorithms. Time series classification is considered the most difficult problem to solve in data mining^23^.

One major challenge in improving the accuracy of proficiency classification based on eye-tracking data is to find and optimize the right features and algorithms. Typical classification techniques used in machine learning are not efficient when dealing with real-valued ordered time-series data, where the temporal ordering and trends of the data are as helpful as the discrete values in providing discriminative information. Such changes are difficult and often impossible to capture using well-known methods, wherein features are individual data points and temporal features are not preserved. This type of problem requires a data primitive that captures temporal changes and temporal criteria for class membership and can be utilized to post analyze the model characteristics responsible for determining class membership^24^.

In recent years, increasingly more problems have been solved using deep learning approaches and neural networks because they require less feature engineering and are thus more suitable if the domain is not well-understood^25^. One of these is the shapelet algorithm originally developed by Ye and Keogh^26^. This algorithm finds shapelets by enumerating all possible candidates and then uses the best shapelet as the splitting criterion at each node of a decision tree. Shapelets are time series sub-sequences that are maximally representative of a class^24,27^. Once the features, such as the Euclidean distances between the series and shapelets, are extracted, they become the input of the classification technique.

As mentioned previously, the development of adequate methods of surgical skill assessment has become critical in a climate of increased awareness of the incidence of iatrogenic injury and medical errors^19^. The integration of eye-tracking techniques and machine-learning algorithms has produced good classification performance in a binary distinction between experts and novices. Researchers have proposed several methods to accurately classify time-series data^28^. Thus, it would be expected that combining deep learning with eye tracking to design a laparoscopic surgical skill assessment tool holds promise not only for a dynamic and flexible mechanism of assessing proficiency but also for simple scalability. The advent of modern deep learning approaches carries the promise of discovering meaningful insights even on massive datasets. Because deep learning models can be generalized from a given set of data^29^, advanced deep learning eye tracking systems will only improve over time as the users' gaze data set grows every time they are used.

However, studies examining differences in laparoscopic surgeons’ skill levels have mostly compared experts and novices, without examining the gaze patterns of participants of intermediate skill. The strategy of using only two groups is problematic because of the lack of information about whether eye movement patterns change in a continuous fashion during the acquisition of more training experience, or whether there is a switching point at which the eye movements of a novice turn into those of an expert. For these reasons, the aim of this study was to examine more levels of training experience and further integrate eye tracking techniques and deep learning algorithms to develop an objective assessment tool for laparoscopic surgical skills.

## Methods

### Ethics

The experiment was reviewed and approved by the Research Ethics Committee of National Taiwan University and compliance with relevant guidelines and regulations. All individual participants in this study gave written informed consent prior to their participation and were free to withdraw from the study without prejudice.

### Participants

Ten right-handed graduate students (eight males and two females) were recruited from the National Taiwan University of Science and Technology to participate in this experiment. The mean age and standard deviation of the participants were 23.2 years and 0.48 years, respectively. All participants had normal or corrected-to-normal vision with no other physical impairments. None were from the medical school and thus none had any prior surgical experience.

### Apparatus

Peg transfer tasks were conducted in a laparoscopic simulation trainer (40 × 25 × 15 cm, length × width × height) developed for the present study. These tasks were performed by holding two 5 mm curved dissector (ENDOPATH Endoscopic Instruments, Dissector, 5DCD, Ethicon, Inc., United States) instruments passing through a rubber diaphragm representing the patient’s abdominal wall, with a pegboard and triangle set on the other side (Peg Board and Triangles, Product No. 50331, Limbs & Things LTD, United Kingdom). The ports of 10 mm in diameter were located at ± 5.7 cm and ± 135° from the center of the rubber diaphragm, and the fulcrum was located approximately halfway up the instrument shaft. The handle of the instrument was set at 80% of the participant’s elbow height, as suggested by Berquer, et al.^30^. A horizontal occluding board was used to prevent direct vision of the instrument displacement during the tasks.

**Figure 1:**
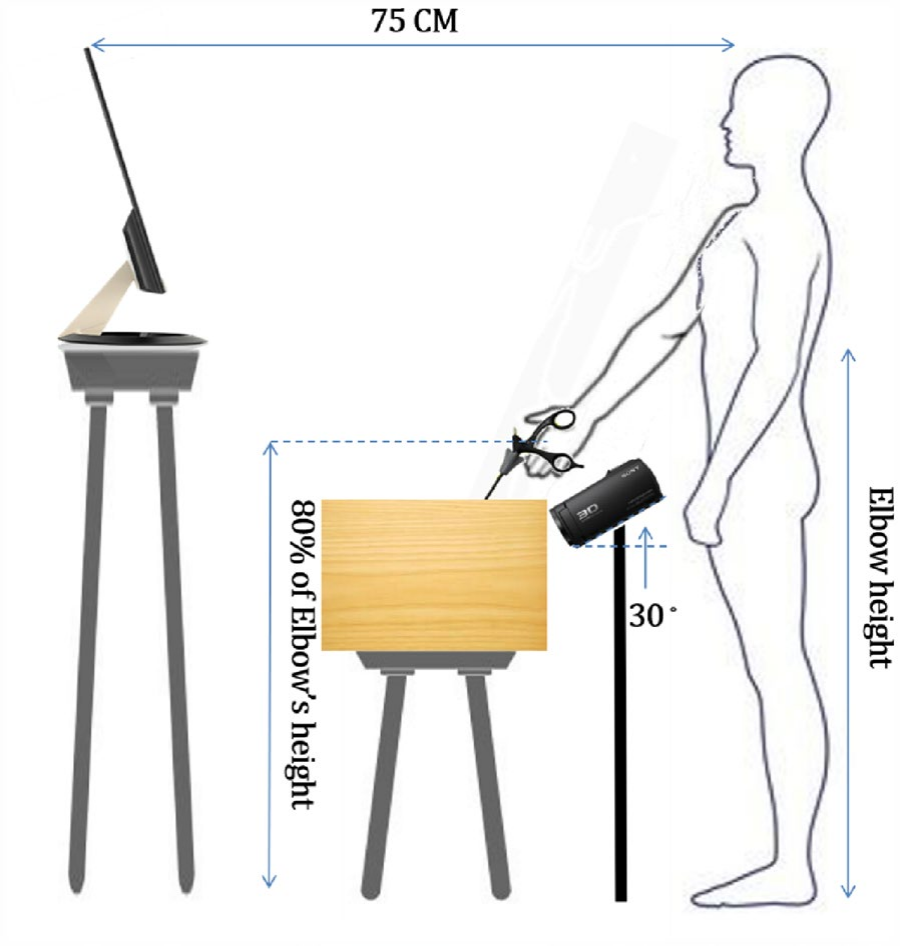
Experimental environment.

The participants controlled the movement of the instrument based on visual feedback from a liquid–crystal display (VZ249H 23.8-inch LCD, ASUS, Taiwan) at a resolution of 1920 × 1080 pixels. A camcorder (HDR-TD30V Full HD Handycam Camcorder, Sony, Taiwan) was set an angle of 30° from the horizontal plane to capture real-time displacement movement in the laparoscopic simulation trainer for synchronous display on the LCD. The viewing conditions were standardized by adjusting the screen height according to each participant’s eye level and maintaining an eccentric viewing angle of 90° and a viewing distance of 75 cm from the participant’s eyes, as shown in Fig. 1.

This study used the Tobii Pro Nano (Tobii Technology, Stockholm, Sweden) remote eye tracker with a sampling rate of 60 Hz mounted on the bottom edge of the LCD to collect the gaze data. To track eye movements, each participant completed a brief eye-calibration process. While standing, participants were asked to observe a moving dot on the eye-tracking monitor. This calibration process took less than 1 min to complete.

### Tasks

**Figure 2:**
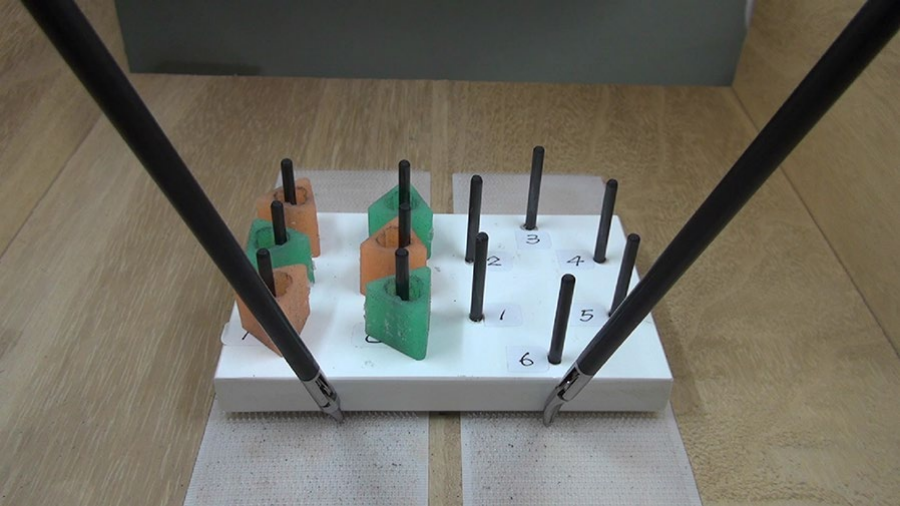
FLS psychomotor task, Peg transfer.

The Fundamentals of Laparoscopic Surgery (FLS) program was developed by members of the Society of American Gastrointestinal Endoscopic Surgeons over the better part of the last decade as an educational curriculum to represent the fundamental cognitive knowledge and technical skills unique to laparoscopic surgery. The peg transfer task is an FLS task designed to develop depth perception and visual-spatial perception in a monocular viewing system, as well as the coordinated use of both the dominant and non-dominant hands^31^. As illustrated in Fig. 2, a series of 6 plastic rings are picked up in turn with a curved dissector from a pegboard on the participant’s left, transferred in space to the other dissector in the right hand, and then placed around a post on the corresponding pegboard on the right. After all of the rings are transferred from the left to right, the process is reversed, requiring transfer from the right to the left hand. The cutoff time is 300 s, and the penalty score is calculated as the percentage of pegs not transferred as a result of being dropped outside the field of view^32–34^.

### Procedure

Before the beginning of each experiment, the objectives and procedures were explained to the participants, and each participant completed a consent form approved by the Research Ethics Committee of National Taiwan University and a simple demographics sheet. Afterward, participants stood in front of the laparoscopic simulation trainer and held one curved dissector in each hand. The position of the dissector handle was adjusted to be close to 80% of the participant’s elbow height to minimize both discomfort and upper arm and shoulder muscle work^30^. The screen height was adjusted according to each participant’s eye level to maintain an eccentric viewing angle of 90°^35^. The participants completed one completed practice trial (transferring all 6 plastic rings in turn from left to right, and then the same process in reverse) before performing the tasks so that they could become familiar with the apparatus and tasks. After a 5-min break, the participants followed the procedures for the peg-transfer task for the first block of five trials. Each participant carried out 10 blocks (50 trials) in total. Only two blocks were performed in one day, with at least a 4-h break between them. During the task, gaze points were recorded by a Tobii Pro Nano eye tracker.

### Design of the eye tracking skill level detection system

**Figure 3:**
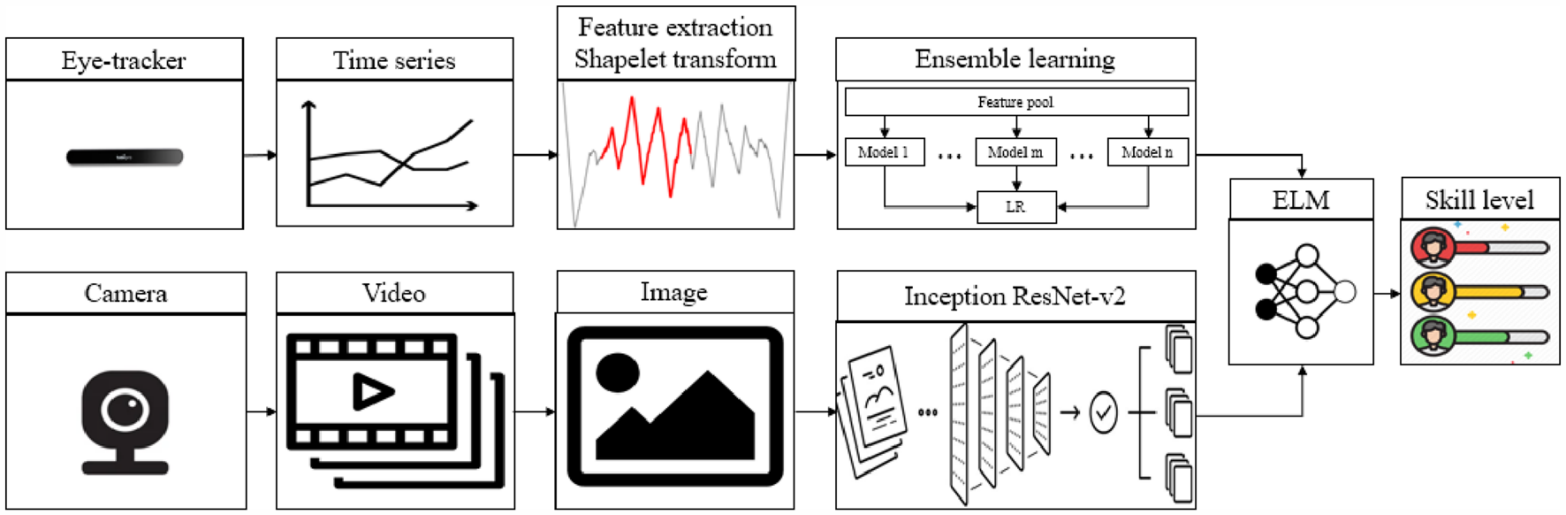
The design process of the eye tracking skill level detection system.

To design our eye tracking task skill level detection system, we developed a classifier with algorithms for our system that could accurately distinguish between different levels of skill following the process shown in Fig. 3. Two types of data sets, including time series data extracted from coordinates of eye fixation and image data from videos, were used to implement and test our proposed skill level detection system. For coordinates of eye fixation, the first step was to input time series data. Second, the data were processed to fit in the shapelet-based model. Third, after the extraction of shapelets from the model, they were classified using a stacked generalization algorithm. As for the videos, a deep learning technique, the convolutional neural network (CNN), was used to classify the image data. Finally, the outcomes from time series and image data were used as inputs to extreme learning machines (ELM) so as to obtain the final results. In the following sections, we explain how we completed each step of the process.

#### Data preprocessing

In the present study, the quality of the captured eye movement data was analyzed. To ensure the quality, participants whose eye movement recordings had a gaze detection rate of less than 80%^36,37^ were removed. The gaze detection rate refers to the percentage of the times that the eyes were correctly detected by the eye tracker for each participant. In this study, the gaze detection rates for all participants met the gaze detection criterion, so no data were removed.

The aim of this study was to develop an assessment method for evaluating skill, so it was necessary to examine more than two levels of skill. For this reason, k-means clustering^38^, an unsupervised and iterative algorithm, was used to split 500 trials into three dissimilar clusters, namely, novice, intermediate, and expert, grouped by peg-transfer task scores. The score of the peg-transfer task was calculated by subtracting both the time required (seconds) and the penalty score (seconds) from a preset cutoff time. The cutoff time was 300 s, and the penalty score was calculated as the number of pegs not transferred when dropped outside of the field of view, multiplied by a time penalty of 17 s^39^.

Since participants finished the experiment in different lengths of time, this study tried to resample the data to overcome this problem by using the Python package *tslearn*. The mechanism behind resampling is as follows: Initially, the first and last numbers are fixed, and then, based on a user-defined size of the output time series, the results return evenly spaced numbers.

As for videos, all the videos were labeled according to their corresponding skill levels. Then the resampling was conducted with different capture rates to extract images evenly from videos of three skill levels and fed into Inception-ResNet-v2.

#### Shapelet transform

The shapelet transform algorithm^40^ is described in Algorithm 1. For each time series, all candidates are generated using the method *generateCandidates*. Thereafter, the distance between the other series and shapelet $S$ are calculated to build the distance list, *D*_*s*_. Distance is calculated as follows:

1 $$Dist\left(\mathrm{S},\mathrm{T}\right)=\genfrac{}{}{0pt}{}{min}{\uprho \in {P}_{l}}(dist(S,\rho ))$$

where ${P}_{l}$ contains all $l$ length subsequences in a series $\mathrm{T}$ and $Dist$ is the Euclidean distance between the same length of shapelet $S$ and $\rho$. When the order list is created, all candidates in the candidate pool are processed by *assessCandidate* to determine the quality of shapelets based on certain quality indicators, such as information gain, moods median and *F*-stat. After that, the shapelets are sorted in descending order based on their quality, and those shapelets with overlapping and those of the lowest quality are removed by *removeSelfSimilar*. The remaining shapelets are added to the shapelet set. Once the best $k$ shapelets have been found, Algorithm 2 is implemented. The importance of transformation is that it provides a way to recall the original data from the feature space by using formula (1). For all shapelets $s$ in $k$, the distance between ${s}_{j}$ and ${T}_{i}$ is calculated, where $j=\mathrm{1,2},\dots ,k$ and ${T}_{i}$ is an instance of dataset **T**. The resulting *k* distances correspond to the distance between a shapelet and the original time series.
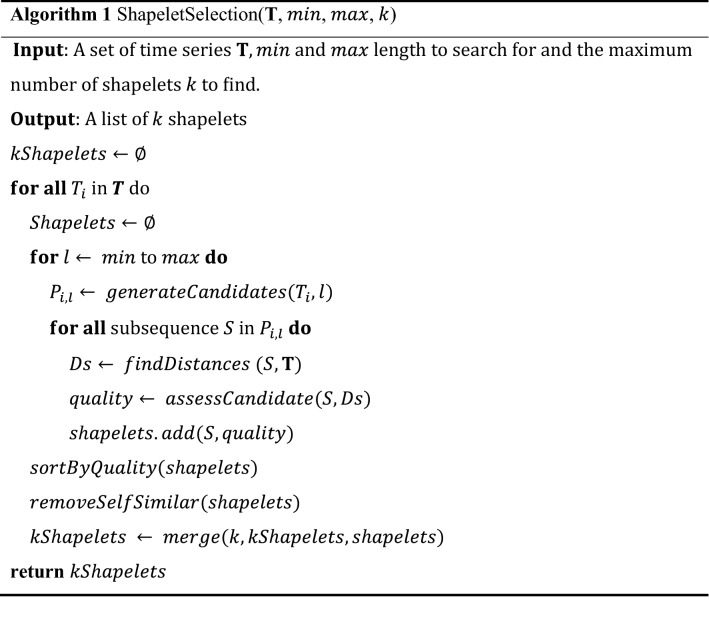

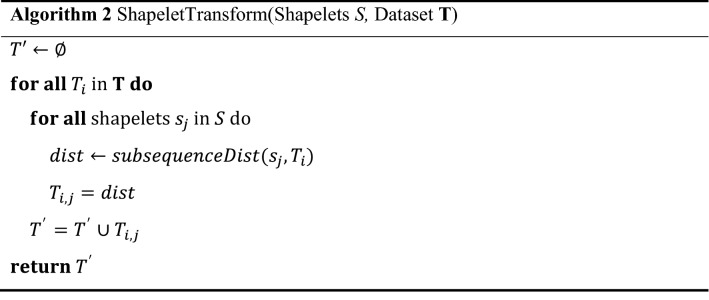


**Figure 4:**
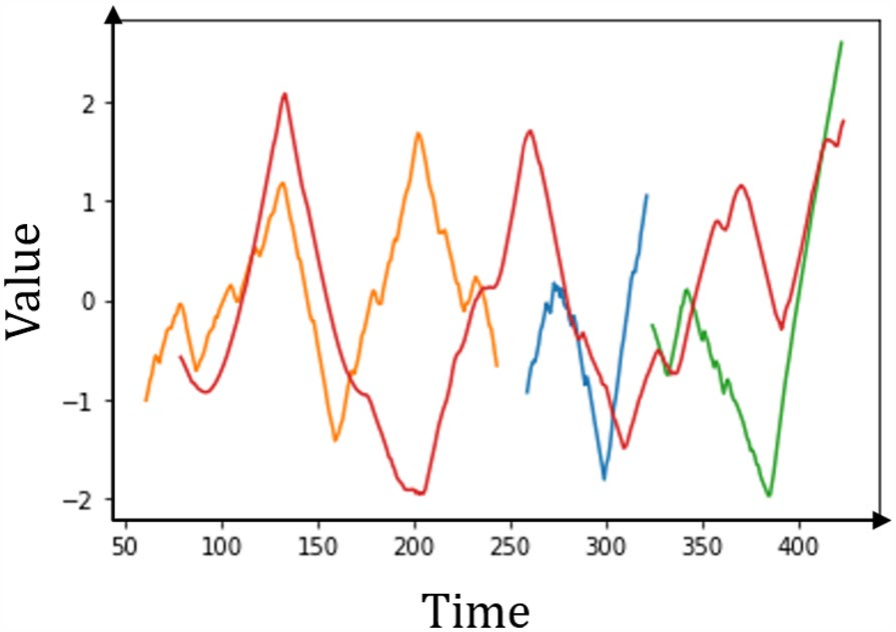
Multiple shapelets extracted from OSULeaf.

**Figure 5:**
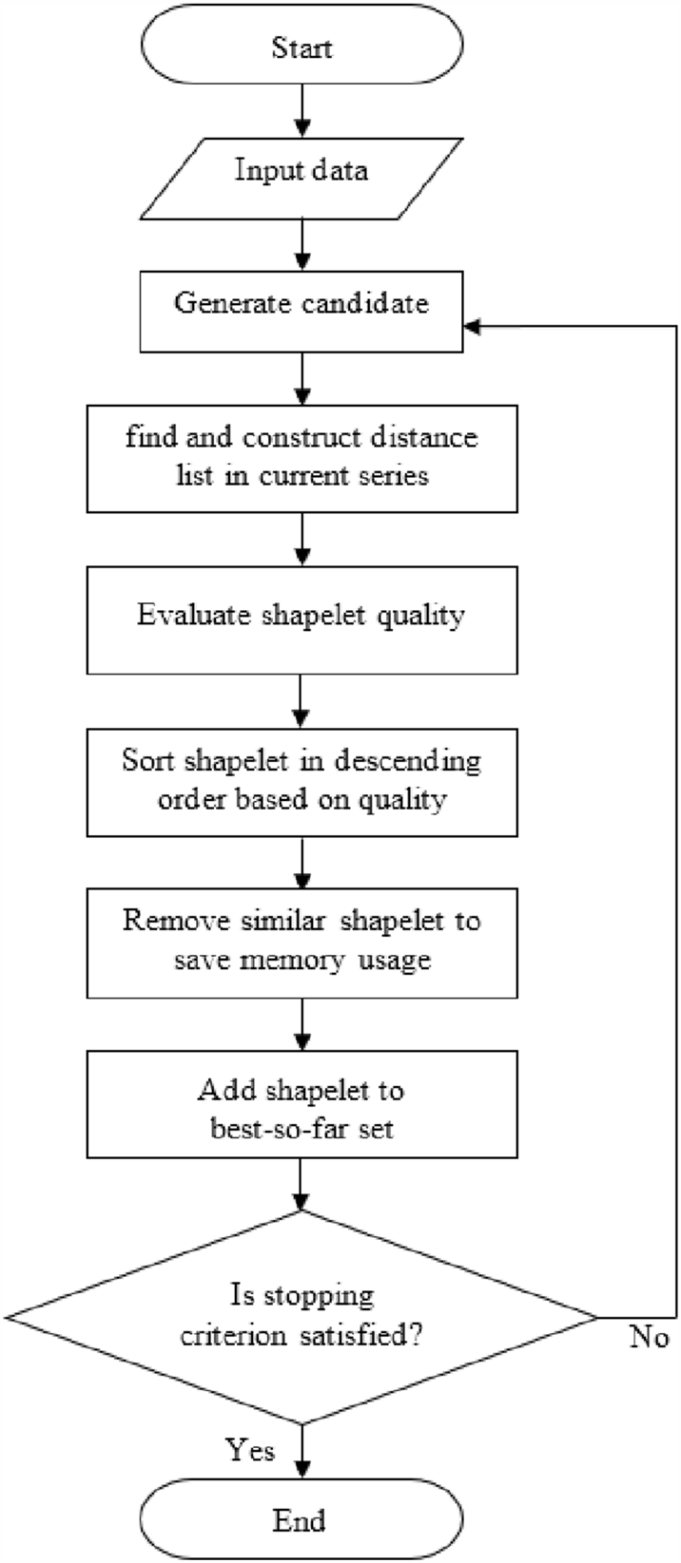
Flowchart of shapelet transform.

For explanation of the shapelet transform algorithm, a simple example is provided as follows. The dataset used is OSULeaf provided by sktime^41^ and consists of one-dimensional outlines of leaves. Series are obtained by image segmentation and boundary extraction. First, all series are the input of the shapelet transform algorithm. Second, candidates for certain series are generated and stored in a candidate pool. After the assessment of the shapelet quality and removal of self-similar shapelets, the shapelets appear as shown in Fig. 4. Multiple shapelets were definitely extracted, so we can use these features and apply any classifier to perform classification. Thus, a shapelet consists of a time series. The number of shapelets extracted depends on the time series itself and the time used to extract the features. The flowchart of shapelet transform of this example is shown in Fig. 5.

#### Ensemble learning

Once the shapelets are all extracted and the transformation is completed, they become the features of the time series. Then stacked generalization, logistic regression, is employed to acquire the best accuracy by borrowing the advantage of heterogeneous classifiers including SVM, Random forests (RF)^42^, Extremely randomized trees (ET)^43^, MLP, AdaBoost^44^, GBDT^45^, and XGBoost^46^. For training the base models, *K*-fold cross validation is conducted. In addition, majority voting is applied to produce the final prediction. Then the classification results for all the base models become the inputs for the ELM model.

To obtain better results, it is necessary to determine the number of trees for the tree ensemble. The number of trees is set to 100 based on Oshiro, et al.^47^. With between 64 and 128 trees, a good balance between processing time and memory usage can be obtained. For AdaBoost, the number of trees is set to 100 and the learning rate is 0.1. For MLP, the number of iterations is 3000, since smaller numbers of iterations do not allow convergence. For SVM, RBF is used as the kernel function. However, since polynomials have more parameters, more time is needed for processing. For XGBoost, the number of trees is set to 100, and the maximum depth of trees is set to 3. The K value in cross-validation varies according to the amount of data; in this study, it was 3 due to the small size of the dataset. For ELM, the weights and bias are randomly assigned initially.

#### Image classification

After preprocessing of the video data, the Inception ResNet-v2^48^ model is used as the CNN algorithm to classify the image data. The video per frame is the input of Inception ResNet-v2. The fully-connected layers are applied, the number of layers is three, and the number of output nodes is equal to the number of classes. In addition, the weights of Imagenet are used. After the extraction of features from the images is completed, all the weights are stored after the model is trained, and predictions are made for new videos in order to test and evaluate the performance. The data are divided into two groups: 2/3 for training and 1/3 for testing.

#### Extreme learning machine

Once the time series and video data have been classified, this study applies ELM to combine the classification results together and in turn to obtain a single final result. This concept is known as data fusion. ELM has been applied in many applications and has shown very promising results^49^. In addition, as a kind of neural network, it has more advantages than the traditional back-propagation neural network.

## Results

### Dataset

The numbers of trials grouped by k-means clustering for three skill levels, namely, novice, intermediate, and expert, were 43, 122, and 335, respectively. A one-way analysis of variance with a significance level of 0.05 was used to determine whether any statistically significant differences existed between the means of the three levels. The test results indicated significant differences in task score (F_2, 497_ = 1192.626, p < 0.001) between the three levels. The mean scores and standard deviations for the novice, intermediate, and expert level were 94.8 (31.9), 160.9 (15.7), and 210.2 (13.2), respectively.

The dataset of eye fixation coordinates acquired from eye-tracking system contained a total of 500 records. Resampling was applied to all data so as to make all time series equal in length; i.e., 16,528 data points. After shapelet transform, about 5 to 10 shapelets were extracted from the data, depending on the algorithm’s search speed. Thereafter, based on the extracted shapelets, they were used as the input of the classifiers. Finally, the output of ensemble learning was the skill level.

For the videos, since participants began developing proficiency after fifteen to twenty iterations, the ratio of novice to intermediate to expert was 1 to 3 to 8. With the help of different capture rates, the final ratio of the different levels was equal. Then the videos per frame were extracted and fed into Inception ResNet-v2 to extract the features from the images. The input resolution of the images was set to 299 × 299 to fit the requirements of the source code, and a total of 70,000 to 80,000 images were fed into deep learning. The number of images depended on the length of each video. The output of the video was the skill level of the operator.

### Ensemble learning

In the present study, ensemble learning was applied to overcome the drawbacks of using only an individual model. All algorithms in the ensemble learning were executed 30 times, and the computational results are shown in Table 1.

**Table 1:** The accuracy of training and testing for single and ensemble models.

Algorithm	Testing accuracy	
	Mean	STD
SVM	0.743	0.055
RF	0.763	0.058
AdaBoost	0.722	0.057
ETC	0.762	0.060
MLP	0.760	0.057
XGB	0.763	0.063
GB	0.764	0.063
Ensemble	0.766	0.052

If a single model was used for eye-tracking data, the mean testing accuracy would fluctuate from the worst of 72.2% for AdaBoost to the best of 76.4% for GB. However, when the ensemble learning was applied, the mean testing accuracy was 76.6% with a lowest standard deviation of 5.2%. This implies that the ensemble method is more stable than the other models.

### Image classification

This study employed the Inception ResNet-v2 model to classify the image data. After 30 executions, this algorithm was able to accurately distinguish three skill levels with accuracy (standard deviation) of 81.2% (2.8%). This result implied that video data can more accurately represent the skill level, for such accuracy is very competitive compared with that of eye-tracking data.

**Figure 6:**
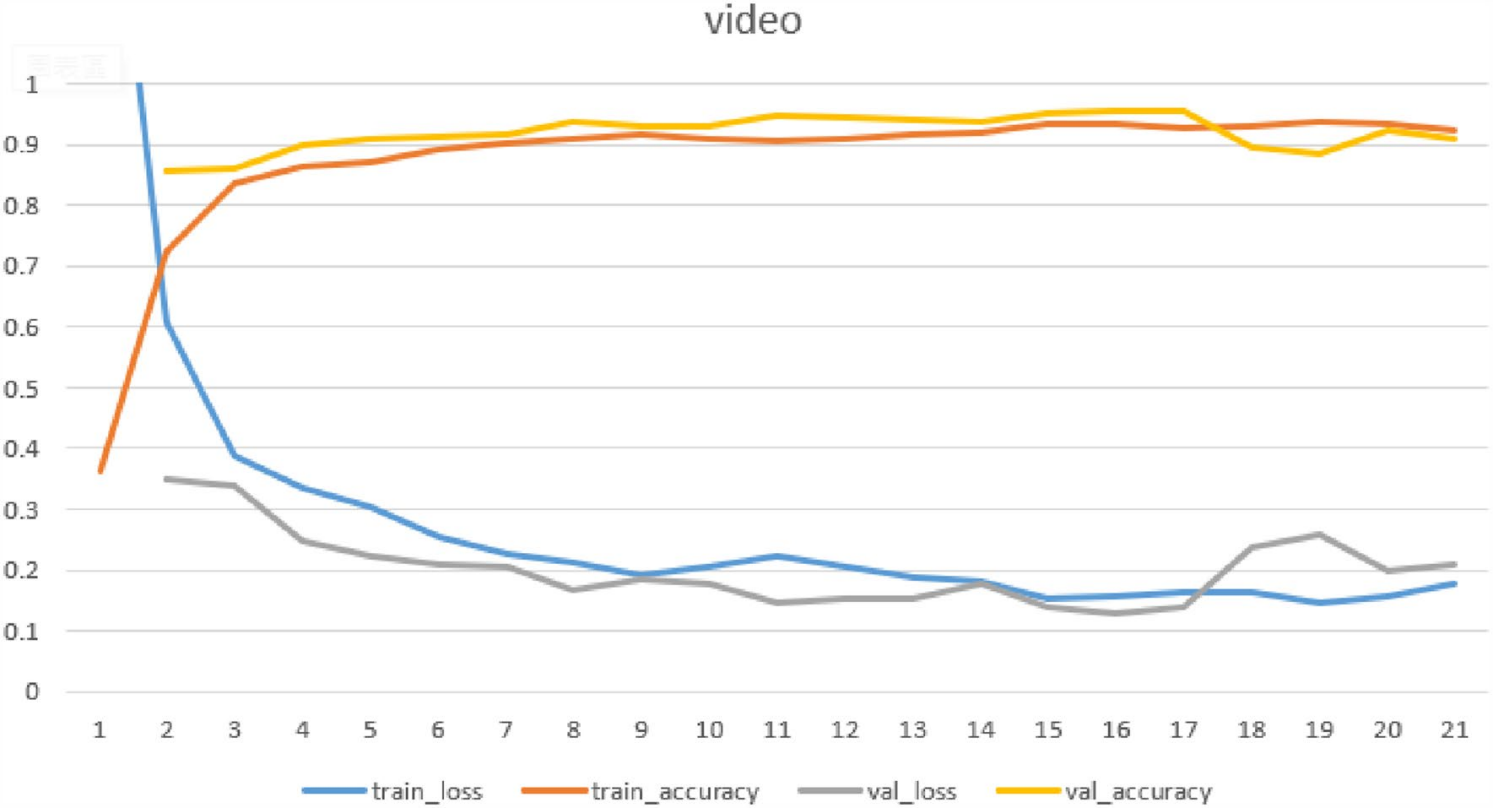
Convergence curve of Inception ResNet-v2.

In addition, Fig. 6 illustrates the convergence curves for Inception ResNet-v2. This figure shows that the algorithm can converge within about 10 iterations.

### Extreme learning machine

Furthermore, this study applied ELM by incorporating the classification results from different types of data, i.e., eye-tracking data and video data, to obtain a single final result. Thus, it was necessary to compare whether or not the accuracy was enhanced. For the parameters of ELM, the activation function was a hyperbolic tangent, the number of hidden nodes was 20, and the number of samples was 377, by default. Moreover, all algorithms were executed 30 times. The mean accuracy and standard deviation were 82.5% and 3.0%, respectively. This result showed that the incorporation of both eye-tracking and video data can yield the best prediction.

In addition, Table 2 displays the confusion matrix of the peg-transfer task. This table reveals that the proposed method can clearly distinguish between novice and expert operators but still has a small chance of misclassifying adjacent levels.

**Table 2:** Confusion matrix of the peg-transfer task dataset.

Label	Prediction		
	Novice	Intermediate	Expert
**Actual**			
Novice	7	6	0
Intermediate	4	15	9
Expert	0	3	82

### Comparing classification algorithms

In the present study, three classifiers, eye fixation coordinates with an ensemble learning approach, video data with a CNN algorithm, and incorporating both data types with an ELM algorithm, were adopted for developing our task skill level detection system. To understand the classification performance between different classifiers, a complete randomized design analysis of variance (ANOVA) was used to investigate the main effects of the classifier on accuracy with a significance level of 0.05. Specific post-hoc comparisons of independent variables were conducted with the Tukey HSD test. The confidence level for statistical significance was set at alpha equal to 0.05.

The ANOVA results revealed significant differences in accuracy between classifiers (F_2,87_ = 24.08, *p* < 0.001). Mean accuracies (Standard Deviation) for the three classifiers of ensemble learning, CNN, and ELM were 76.0% (5.2%), 81.2% (2.8%), and 82.5% (3.0%), respectively. Further post hoc Tukey HSD test results showed that the accuracy was significantly different between the three classifiers.

## Discussion

This study examined more levels of training experience and further integrated an eye tracking technique and deep learning algorithms to develop an objective assessment tool of laparoscopic surgical skill. Our study extracted features from eye-tracking data collected during the performance of a peg transfer task to investigate their capacity for detecting levels of skill.

It the present study, ten nonmedical students were recruited to perform the peg transfer tasks. Then k-means clustering^38^ was used to split 500 trials into three dissimilar clusters grouped by peg-transfer task scores. The mean scores obtained in the present study for the novice, intermediate, and expert level were 94.8, 160.9, and 210.2, respectively, which are substantially consistent with the results of Maddox, et al.^50^ and Derossis, et al.^34^. In the study by Maddox, et al.^50^, the mean scores for the novice (1st and 2nd year medical students), intermediate (PGY 2–5 urology residents), and expert (urology department attending physicians) levels were 95, 164, and 190, respectively. This finding provides support for applying our results to surgeons with different levels of experience. Furthermore, the score for the expert in the present study (210.2) was consistent with that (208) obtained from general surgery residents at the PGY3 level who had a total of seven repetitions in a study by Derossis et al.^34^. This finding implies that the performance on the most fundamental peg-transfer task of nonmedical students would be similar to that of medical experts, as long as sufficient practice is allowed.

Since participants began developing proficiency after fifteen to twenty trials, and the ratio of novice to intermediate to expert was 1 to 3 to 8. With a relatively unbalanced sample, our results showed that the approach combining the features of visual fixation coordinates and images could classify three different levels of skill with 82.5% accuracy. Based on our findings, the time series performance was affected by the time setting from the users. This suggests that longer times would produce better results. Although the statistical analysis of ensemble learning is not superior to those of other methods, it can overcome the drawback of the dependency of only using a single model.

Although the proposed method has demonstrated a very promising performance for classifying skill levels of trainees, this study has several limitations. First, our results were limited by the task context, which in our study was an FLS peg transfer task. Future studies will be needed to use more demanding FLS tasks to verify and extend our results. Second, the results were limited to a fairly specific population, namely graduate students in a management school. For validation purposes, surgeons having different levels of skill and amounts of laparoscopic surgery experience should be recruited in future studies to provide a deeper understanding of the relationship between surgical skills and eye parameters^11^. Third, the method of extracting features from time series is time-consuming. Practitioners studying the extraction of features may consider applying a more powerful algorithm on time series for feature extraction to produce more accurate results and reduce computational time. In recent years, many researchers have focused their interest on transforming time series into matrices and applying deep learning techniques. With a similar concept, the current time series can also be transformed and a novel CNN can be applied for classification. Finally, a class-imbalance dataset obtained from k-means clustering might result in a greater focus on classification of the majority group while misclassifying the minority group. For this reason, methods for solving the class-imbalance problem proposed at both the data and algorithmic levels will be need to be applied in future studies.

## Conclusion

Our study extracted features from eye-tracking data and images collected during a peg-transfer task to investigate their capacity for detecting skill levels. With a relatively small sample, our results showed that combining the features of fixation coordinates and images allowed accurate classification of trainees of various skill levels. This result not only proposes a possibility of using deep learning algorithms with gaze-based and image features as a method of assessing surgical skills but also provides valuable insights as well as motivation for future studies focused on designing skill detection systems. Finally, it is recommended that more demanding FLS tasks, surgeons of different levels of skill, and a larger participant sample should be examined with the DL approach by combining features obtained from different modalities to evaluate how these objectively measured features could contribute to the assessment of laparoscopic skill.

## Data Availability

The datasets generated and analyzed during the current study are available from the corresponding author on reasonable request.
